# Were sea level changes during the Pleistocene in the South Atlantic Coastal Plain a driver of speciation in *Petunia* (Solanaceae)?

**DOI:** 10.1186/s12862-015-0363-8

**Published:** 2015-05-20

**Authors:** Aline MC Ramos-Fregonezi, Jeferson N Fregonezi, Gabriela B Cybis, Nelson JR Fagundes, Sandro L Bonatto, Loreta B Freitas

**Affiliations:** Laboratory of Molecular Evolution, Department of Genetics, Universidade Federal do Rio Grande do Sul, P.O. Box 15053, Porto Alegre, Brazil; Department of Statistics, Universidade Federal do Rio Grande do Sul, P.O. Box 15080, Porto Alegre, Brazil; Genomic and Molecular Biology Laboratory, Pontifícia Universidade Católica do Rio Grande do Sul, Ipiranga 6681, 90610 001 Porto Alegre, RS Brazil

**Keywords:** Genetic diversity, *Petunia*, Phylogeography, Plant speciation, Pleistocene, Refuge, South Atlantic Coastal Plain

## Abstract

**Background:**

Quaternary climatic changes led to variations in sea level and these variations played a significant role in the generation of marine terrace deposits in the South Atlantic Coastal Plain. The main consequence of the increase in sea level was local extinction or population displacement, such that coastal species would be found around the new coastline. Our main goal was to investigate the effects of sea level changes on the geographical structure and variability of genetic lineages from a *Petunia* species endemic to the South Atlantic Coastal Plain. We employed a phylogeographic approach based on plastid sequences obtained from individuals collected from the complete geographic distribution of *Petunia integrifolia* ssp. *depauperata* and its sister group. We used population genetics tests to evaluate the degree of genetic variation and structure among and within populations, and we used haplotype network analysis and Bayesian phylogenetic methods to estimate divergence times and population growth.

**Results:**

We observed three major genetic lineages whose geographical distribution may be related to different transgression/regression events that occurred in this region during the Pleistocene. The divergence time between the monophyletic group *P. integrifolia* ssp. *depauperata* and its sister group (*P. integrifolia* ssp. *integrifolia*) was compatible with geological estimates of the availability of the coastal plain. Similarly, the origin of each genetic lineage is congruent with geological estimates of habitat availability.

**Conclusions:**

Diversification of *P. integrifolia* ssp. *depauperata* possibly occurred as a consequence of the marine transgression/regression cycles during the Pleistocene. In periods of high sea level, plants were most likely restricted to a refuge area corresponding to fossil dunes and granitic hills, from which they colonized the coast once the sea level came down. The modern pattern of lineage geographical distribution and population variation was established by a range expansion with serial founder effects conditioned on soil availability.

**Electronic supplementary material:**

The online version of this article (doi:10.1186/s12862-015-0363-8) contains supplementary material, which is available to authorized users.

## Background

The severe climatic oscillations that occurred during the Pleistocene produced major changes in species distribution and consequently in their genetic diversity. These changes strongly impacted the vegetation at different latitudes and longitudes [1,2]. Some species became extinct over much of their range, some dispersed to new locations, and others survived in refuges and then expanded again; all of these events could have occurred repeatedly during the period [3,4]. Fragmentation, contraction, and expansion of species’ distribution occurred during the Quaternary climate fluctuations and have been suggested as an explanation for the current patterns of genetic diversity found in different lineages [5-7].

The response of vegetation in the Southern Hemisphere to the glacial-interglacial oscillations has been substantially different from that of the Northern Hemisphere [8,9]. Studies from several taxa have reported deep phylogeographic structures in South America dating to the Neogene, possibly as a consequence of old marine transgressions and the uplift of the Andes (for review see [10]). Nevertheless, genetic consequences of recolonization after the last glacial maximum (LGM) ca. 20 000 years before the present have also been described in species from South America (*e.g.,* [4,10-12]).

Quaternary climatic changes also led to variations in sea level, which was lower during glacial periods and higher during interglacial periods [13]. Periodical transgressions of 100 m above the present level have been reported for the Quaternary [14,15], and some studies [16,17] have indicated that sea level changes during the Quaternary played a significant role in the generation of marine terrace deposits in the South Atlantic Coastal Plain (SACP). Studies along the east-northeast Atlantic Coast in South America have shown that during high sea level, barrier island lagoon systems were the dominant mode of sedimentation (reviewed by [18]). In the South Atlantic, these systems resulted in a geological formation known as the SACP, which consists of four barrier lagoon depositional systems (Barrier I, II, III and IV) representing the sedimentary record of a marine transgression: three from the Pleistocene and one from the Holocene, dating to 400 000, 325 000, 125 000, and 7000 years before the present, respectively [19].

The main consequence of the increase in sea level was local extinction or population displacement, such that coastal species would be found around the new coastline. Thus, for coastal species, estuaries and slopes of the mountains may have acted as refuge areas during interglacial periods. Under the refuge hypothesis, one could expect to find evidence of high genetic diversity in areas of stability and lower diversity and the molecular signatures of recent range expansion in the species in unstable, recently recolonized regions [1,20]. The refuge theory has been widely tested for tropical Neotropical biomes [10], but its general relevance for non-forested biomes has been less explored [10,12].

If refuge areas were important in the SACP during the Pleistocene, it should be possible to find a correlation between genetic diversity and putative ancient refuge areas (see [9,20]). More specifically, one would expect to find areas of high genetic diversity and no signal of recent population growth in refuge areas, whereas recently colonized habitats would have lower genetic diversity and signals of population expansion. Moreover, the age of genetic lineages should be congruent with geographical distribution, and population expansion must postdate SACP origin.

**Figure 1:**
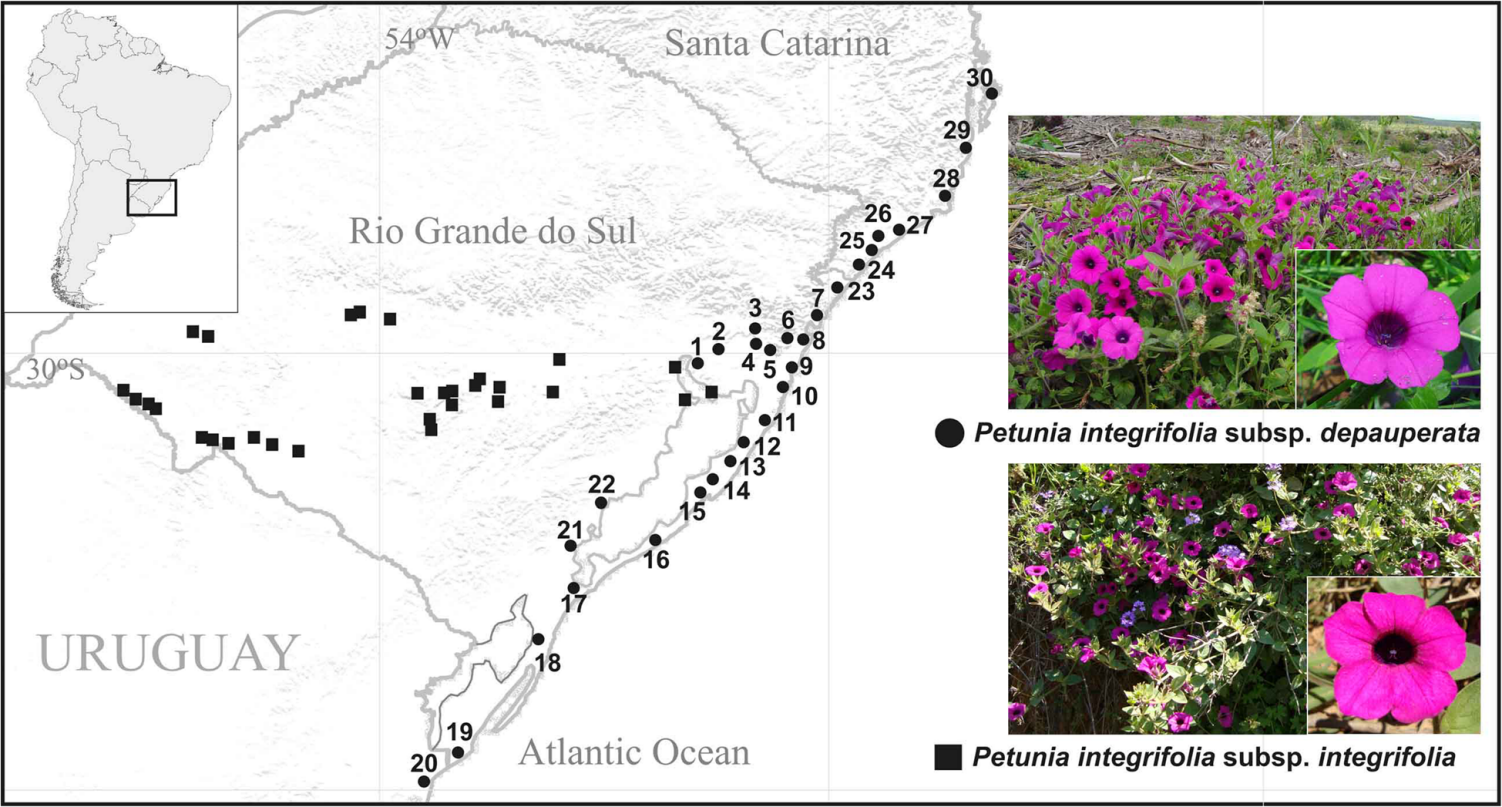
Plant material. Map of the sampling sites for the two *Petunia integrifolia* subspecies, where black circles (numbers 1 to 30) represent *Petunia integrifolia* ssp. *depauperata* samples and black squares correspond to *Petunia integrifolia* ssp. *integrifolia.* More information on collection sites is available in Additional file 2. Right side: representatives of two *Petunia integrifolia* subspecies, general view of individual and flower detail.

*Petunia integrifolia* is a bee-pollinated species that presents purple flowers [21] and is part of the short tube group [22,23] and lowland clade of *Petunia* [24]. According to Stehmann and Bohs [25], this species comprises two subspecies (Figure 1): *P. integrifolia* ssp. *integrifolia* and *P. integrifolia* ssp. *depauperata* (Fries) Stehmann and Semir. The former is widespread in the Pampas region (Uruguay, part of Argentina, and southern part of Rio Grande do Sul, Brazil), while the latter is endemic to the SACP, from Florianópolis (Santa Catarina, Brazil) to Chuí (border between Brazil and Uruguay). Each subspecies represents a different evolutionary lineage based on sequences of the internal transcribed spacers of ribosomal nuclear DNA and plastid DNA [26].

In this work, we used a phylogeographic approach to characterize the genetic diversity of a comprehensive sample of *P. integrifolia* ssp. *depauperata*, looking for geographical patterns of genetic variation and lineage distribution that could have emerged during the Pleistocene. Our main aim was to investigate the effects of sea level changes and the putative role of refuge areas for *P. integrifolia* ssp. *depauperata* using sequences from two plastid intergenic spacers. We chose these genetic markers because they have been successfully used in phylogeographic studies in *Petunia* [24,26-30] and in a closely related genus, *Calibrachoa* Cerv. [31].

## Results and discussion

### Sequence analysis

A general characterization of each marker is shown in Table 1. For *P. integrifolia* ssp. *depauperata*, the concatenated alignment resulted in 1120 bp, with 25 variables sites and 19 parsimoniously informative sites, resulting in 26 haplotypes. For *P. integrifolia* ssp. *integrifolia*, the concatenated alignment resulted in 1071 bp, with 15 variable sites and seven parsimoniously informative sites, resulting in 13 haplotypes. Variable and informative indels produced the discrepancies between the complete alignment and individual sequence length ranges. Despite the great morphological similarity and their taxonomic status, these two taxa did not share haplotypes, which is surprising because haplotype sharing is widely reported among *Petunia* species [24,28]. Another example of haplotype sharing is the three *P. axillaris* (Lam.) Britton, Sterns and Poggenb subspecies, which cannot be identified based on these plastid sequences alone [29].

**Table 1:** **Characterization of plastid markers used in this work**

**Taxa**	**Molecular Marker**	**Length (bp)**	**%GC**	**V**	**Substitutions**	**Indels**	**I (%)**
*P. integrifolia* ssp*. depauperata*	*trnH psbA*	457	27	17	14	3	11 (64.7)
	*trnG trnS*	663	30	8	6	2	8 (100)
	**Total**	1120	57	25	20	5	19 (76)
*P. integrifolia* spp*. integrifolia*	*trnH psbA*	415	27	8	7	1	2 (25)
	*trnG trnS*	656	30	7	5	2	5 (71.4)
	**Total**	1071	57	15	12	3	7 (46.7)

bp – base pairs; V – number of variable sites; I – number (proportion) of parsimoniously informative variable sites; total – both markers concatenated.

As described in Table 2, the molecular diversity presented for *P. integrifolia* ssp. *depauperata* was *h* = 0.77 ± 0.01 and *π* (%) = 0.13 ± 0.09. All parameters were in accordance with other data obtained for other *Petunia* species in different surveys, despite the wide difference in geographic distribution range and sample sizes among species. In a previous study including *P. integrifolia* ssp. *depauperata*, Longo et al. [26] found only nine haplotypes and similar diversity indices using far fewer individuals. *P. exserta* Stehmann, a species with a restricted geographic distribution, was analyzed using the same genetic markers and a similar sample size and presented only six haplotypes but similar nucleotide diversity [28]. The three subspecies of *P. axillaris,* that together cover a geographic range larger than that of *P. integrifolia* ssp. *depauperata*, presented 35 haplotypes, but nucleotide and haplotype diversity values [29] were close to those reported in the present study.

**Table 2:** **Summary statistics obtained for the three haplogroups and for the whole sampled sequences of** ***Petunia integrifolia*** **ssp.** ***depauperata***

**Haplogroup**	**Individuals (n)**	**Haplotypes (n)**	**Haplotype diversity (sd)**	**Nucleotide diversity % (sd)**	**Tajima’s D**	**Fu’s** ***F***_**S**_
Southern	126	11^#^	0.34 (0.05)	0.03 (0.03)	**-1.92**	**-11.30**
Center	63	7	0.66 (0.03)	0.08 (0.06)	-0.49	-2.07
Northern	100	7	0.27 (0.05)	0.04 (0.04)	-1.40	**-4.09**
**Total**	289	25^#^	0.77 (0.01)	0.13 (0.09)	-1.31	**-16.46**

^#^H19 not included; n – number; sd – standard deviation; bold values indicate significant neutrality test D (P < 0.05) and *F*_S_ (P < 0.02).

### Phylogenetic relationships and molecular dating

**Figure 2:**
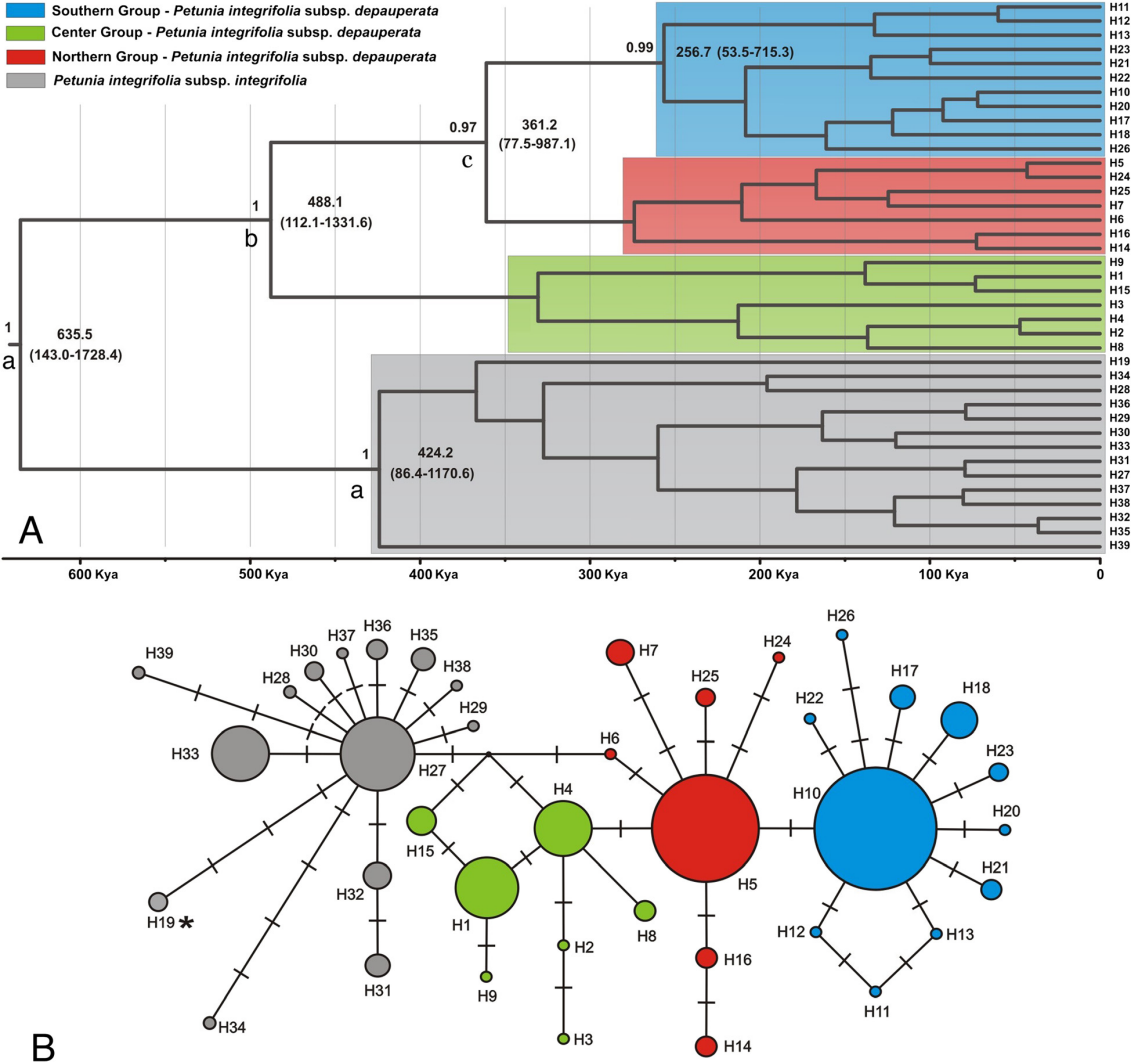
Evolutionary relationships among haplotypes of *Petunia integrifolia* subspecies. **(A)** Bayesian phylogenetic tree with posterior probabilities (PP > 0.9) shown above the branches and ages indicated below branches for selected nodes in thousands of years (Kya). Confidence intervals are presented in parenthesis; selected nodes marked with “a” indicate the higher probability of Most Recent Common Ancestor origin in *Petunia integrifolia* ssp. *integrifolia* group and “b” and “c” indicate Center and Southern Groups of *Petunia integrifolia* ssp. *depauperata,* respectively (see Table xx for probabilities); **(B)** Haplotype median-joining network. Sizes of the circles are proportional to the overall frequency of the haplotypes, and the color within each circle represents different genetic groups, according to the colors on the left and the Bayesian inference. Crossed lines represent inferred differences between haplotypes. The haplotype marked by asterisk (H19) belongs to *Petunia integrifolia* spp. *depauperata* population 17 (more details in the main text).

The 39 different haplotypes of *P. integrifolia* were used in the network and phylogenetic analyses (Additional file 1). The median-joining network divided the sample into four major haplogroups (Figure 2). The first three haplogroups were composed of haplotypes found in individuals of *P. integrifolia* ssp. *depauperata* (H1 to H26) and will be further discussed below. The last haplogroup contains all haplotypes of *P. integrifolia* ssp. *integrifolia* individuals (numbers 27 to 39). Haplotype H19 (identified by an asterisk in Figure 2) was an exception, grouping in the same haplogroup as *P. integrifolia* ssp. *integrifolia* haplotypes but appearing in two individuals from population 17 of *P. integrifolia* ssp. *depauperata*. We cannot discard the possibility of hybridization between these species, but natural hybridization is rare in *Petunia* and is limited to *P. exserta* and *P. axillaris* [28]. It is most likely that these individuals considered as *P. integrifolia* ssp. *depauperata* were misidentified during collection because the respective population is located at the limit of the taxa distribution. The haplogroup of *P. integrifolia* ssp. *integrifolia* was separated from *P. integrifolia* ssp. *depauperata* haplogroups by two mutational steps. This fact makes these subspecies suitable for gene studies related to adaptations to osmotic and saline stresses, which could be involved in the differentiation process in this taxa complex.

The three haplogroups of *P. integrifolia* ssp. *depauperata* had a striking geographical structure (Figure 2), with one haplogroup distributed in the central part of the species distribution (Center Group), one in the northern part (Northern Group), and the last distributed in the southern part (Southern Group). Haplotypes from the Center Group were observed in individuals from a region of a fossil dune environment and granitic hills that were not affected by changes in the sea level during the Pleistocene [31,32] and were reminiscent of the oldest sea level transgression/regression cycle (Barrier I; [19]). Haplotypes from the Northern Group were distributed in populations localized in regions associated with Barrier II [19], which is younger than Barrier I. Finally, haplotypes from the Southern Group occurred in a region corresponding to the more recent Barriers III and IV [19]. Notably, the Northern Group was separated by one mutational step from the central haplogroup, while the southern haplogroup was separated from the northern haplogroup by one mutational step. The northern and southern haplogroups exhibited a marked star-like shape, which might indicate a population expansion.

Different populations shared the four most frequent haplotypes (H1, H4, H5, and H10). Only six populations were monomorphic, and 18 haplotypes were exclusives (Additional file 2). In general, neighbor populations shared haplotypes. Sharing ancestral polymorphisms is common in *Petunia* species populations [24,28,29] and *Calibrachoa* [30,33], independent of the geographic distance between the populations. However, neighbor populations may also share haplotypes due to gene flow mediated by pollen and seeds between these populations because the plastid is maternally inherited (through seeds) in *Petunia* species [34]. The nearly continuous geographical distribution of *P. integrifolia* ssp. *depauperata* along the coast may facilitate gene flow either due to higher pollinator displacement or due to seed dispersal caused by the strong winds in the SACP [35,36].

The Bayesian phylogenetic tree displayed two main clades with maximum support values corresponding to *P. integrifolia* subspecies and a divergence time of approximately 635 thousand years ago (Kya; Figure 2 top). The *P. integrifolia* ssp. *depauperata* clade showed the same three haplogroups previously identified in the evolutionary network (Figure 2 bottom). The Center Group was sister to the group formed by individuals from the Northern and Southern Groups. The Northern and Southern Groups diverged from one another ca. 361 Kya, whereas both diverged from the Center Group ca. 488 Kya. These ages are broadly compatible with the estimates of ages for SACP origin and the associated depositional systems [19], which suggest that the diversification of these haplogroups was possible as soon as new habitat was available for plant colonization in 400 kya (Barrier I), 325 kya (Barrier II), 125 kya (Barrier III), and 7 kya (Barrier IV), respectively.

The striking geographic structure of these clades makes the phylogeographic reconstruction very clear for most nodes of the tree (Figure 2), the only exceptions for which the reconstruction is not obvious are the root of the tree and some of the nodes closer to the root (reconstructions marked by letters in Figure 2). Table 3 presents the posterior probabilities for these reconstructions. The scenario with highest posterior probability puts the most recent common ancestor (MRCA) of the whole clade in the region currently occupied by subsp. *integrifolia*, and subsequent migration of the lineage-giving rise to the *depauperata* subspecies to the central region. Our estimates are not clear, however, regarding the MCRA of the populations currently in the southern and northern regions, with high posterior probability of being in either region (Table 3). We explored Bayes factors to find significant migrations between geographic locations, however due to some topographic uncertainty at the most recent nodes of the tree and the few inferred migrations along the tree, most of these results were non-significant.

**Table 3:** **Posterior probabilities for the most recent common ancestor in phylogeographic reconstruction**

**MRCA**	**Southern**	**“integrifolia”**	**Center**	**Northern**
Root	0.23	**0.33**	0.26	0.18
*P. integrifolia* ssp*. integrifolia*	0.15	**0.82**	0.02	0.01
*P. integrifolia* ssp*. depauperata*	0.29	0.16	**0.35**	0.20
South/North	**0.42**	0.07	0.10	**0.41**

The phylogeographic model assumes constant migration rates throughout evolutionary history. While this is clearly not the case for our analyses because some of the regions were submerged and thus unavailable for plant migration, the results highlight our hypothesis of a refuge in the center of distribution and posterior migration to the north and to the south. Even without informing the model that the southern and northern portions of the SAPC were not available habitats early in their evolutionary history, we infer the origin of these populations of *P. integrifolia* ssp. *depauperata* to be in the western regions, from *P. integrifolia* ssp. *integrifolia* individuals, and migration to the coastal regions to only happen in more recent times (Figure 2).

### Population structure and spatial genetic analysis

A two-level AMOVA (i.e., subpopulations vs. the total population) resulted in an estimate of 66% of the total genetic variance found among populations, while the remaining 34% of total genetic variation was found within populations (Table 4). The Mantel test indicated a non-significant association (P = 0.2) between genetic and geographic distances for populations, which may suggest that the genetic differences among populations reflect the underlying genealogical structure of the plastid spacers more strongly than gene flow between neighbor populations.

**Table 4:** **Analysis of molecular variance (AMOVA) for the** ***Petunia integrifolia*** **ssp** ***. depauperata*** **estimated using two hierarchical models: two-level model includes only populations and three-level model includes all populations distributed in the three haplogroups observed**

**Model**	**Source of variation**	**d.f.**	**Sum of squares**	**Variance components**	**Variation (%)**	**Statistics**
Two levels	Among populations	29	152.9	0.516	65.6	
	Within populations	261	70.7	0.270	34.4	
	**Total**	290	223.6	0.787		*Φ*_*ST*_ = 0.656*
Three levels	Among groups	2	122.5	0.645	64.2	*Φ*_*CT*_ = 0.642*
	Among populations within groups	27	30.4	0.088	8.8	*Φ*_*SC*_ = 0.246*
	Among populations	261	70.7	0.270	27	*Φ*_*ST*_ = 0.730*
	**Total**	290	223.6	1.004		

The significance of each *Φ* statistic was tested through 1000 permutations at the appropriate hierarchical level. *P < 0.0001.

**Figure 3:**
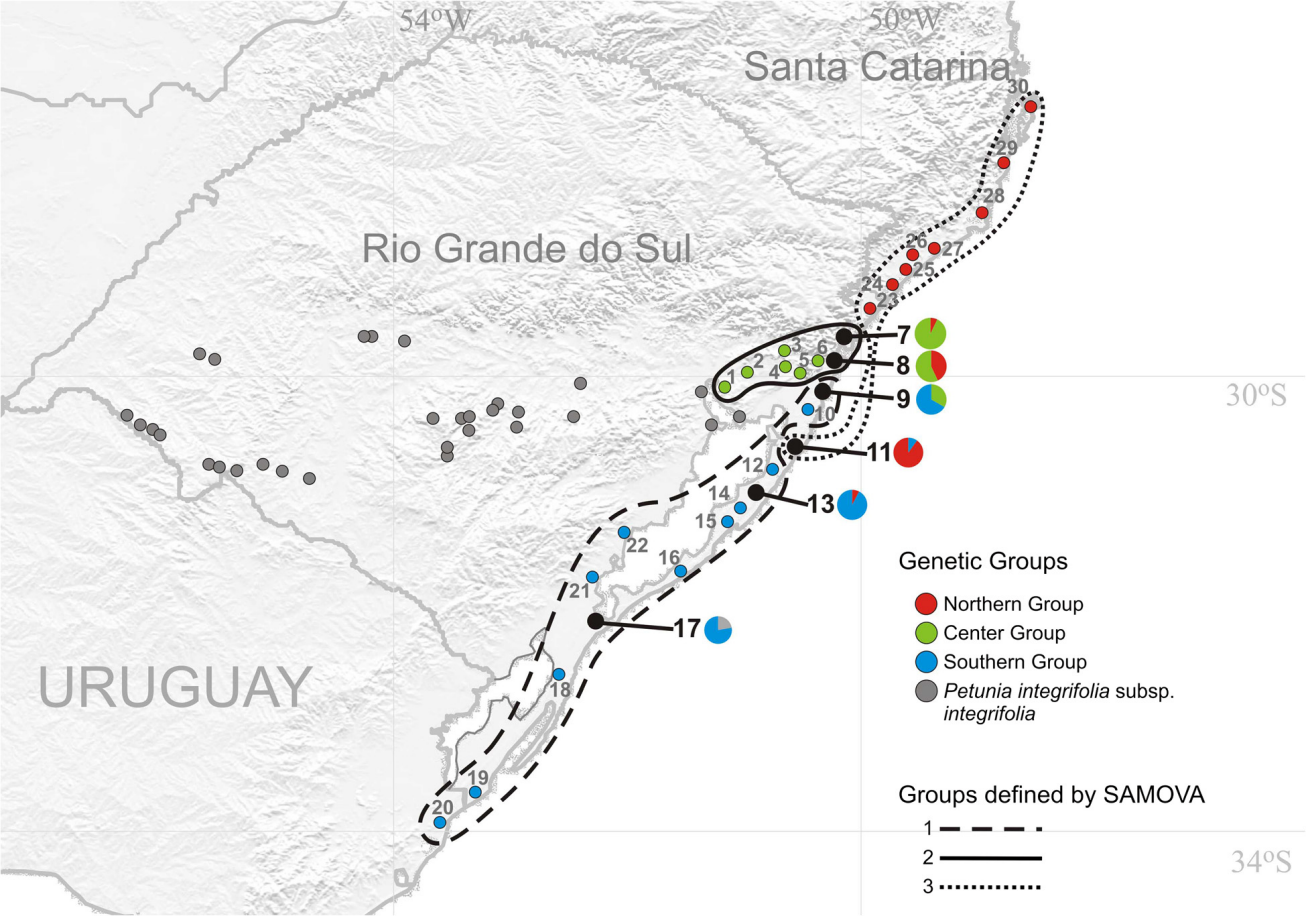
Geographical groups identified by SAMOVA analyses. Each group is indicated by a border style. The colors displayed in the populations correspond to genetic groups found in the phylogeny and the median-joining haplotype network. The gray dots indicate *Petunia integrifolia* ssp. *integrifolia* populations. The black dots highlighted with larger fonts are the populations with haplotypes from different genetic groups. Lines represent SAMOVA groups according the legend.

The Samova analysis suggested that three groups (K = 3) maximize the genetic structure in the among-group level (Φ_*CT*_ = 0.642; P < 0.001) (Figure 3). For K ≥ 4, Samova found similar or lower Φ_*CT*_ values. Populations 1-8 formed Samova’s Group 2 (solid line). Most of these populations only had haplotypes from the Center Group, but populations 7 and 8 also presented, in low frequency, haplotypes H5 and H14 from the Northern Group, respectively. Populations 9-10 and 12-22 formed Samova’s Group 1 (dashed line). Most of these populations only had haplotypes from the Southern Group, except for population 9 (two individuals presenting haplotype H4, from the Center Group), population 13 (one individual presenting haplotype H5, from the Northern Group), and population 17 (two individuals presenting haplotype H19, which is more related to *P. integrifolia* ssp. *integrifolia*). Finally, populations 11 and 23-30 formed Samova’s Group 3 (dotted line). Populations 23-30 only had haplotypes from the Northern Group. The presence of population 11 in SG3, however, is more difficult to explain. This assignment may have occurred because this population had a high frequency of haplotypes H5 and H16 from the Northern Group (but also haplotype H10, from the Southern Group), despite this population being geographically closer to SG1. However, H5 was already present in another population from SG2 (Pop 7), though with minor frequency. Thus, population 11 may actually be part of SG1, but due to stronger drift or founder effect, a higher frequency of Northern Group haplotypes assigns it to SG3. More genetic markers will be necessary to clarify this issue. Notably, the geographical limits of these groups do not match the known putative actual or historical barriers (rivers or other geographic discontinuities) proposed as drivers of population structure of *Calibrachoa heterophylla* (Sendtn.) Wijsman from the same geographic distribution [30].

### Demographic analyses

Fu’s *F*_**S**_ value was negative and significant (-16.5, P < 0.02), whereas Tajima’s D was also negative but marginally non-significant (-1.31, P = 0.05) for *P. integrifolia* ssp. *depauperata*. Tajima’s D and Fu’s *F*_s_ are classical neutrality tests used to assess population demographic history. Both assume that populations have been in mutation–drift balance for a long period of time [37]. Negative values in both tests are indicative of a demographic expansion. Together with the star-like shape observed in the networks, particularly for the Northern and Southern Groups, this may suggest a population expansion associated with SACP colonization.

The Northern group presented the lower haplotype diversity (*h* = 0.27) and a significant Fu’s *F*_**S**_ (-4.09, P < 0.02), while the Southern group displayed the lower nucleotide diversity (*π* = 0.03) and significant and negative values for both neutrality tests (Tajima’s D = -1.92, P < 0.05; Fu’s *F*_**S**_ **=** -11.30, P < 0.02) (Table 2).

The higher genetic diversity and lack of indication of population growth are perfectly compatible with Center group representing a refuge for *P. integrifolia* ssp. *depauperata*. However, the smaller genetic diversity and signals of population expansion suggest that Southern and Northern groups represent distinct events of range expansion from the refuge as long as SACP became available for this taxon.

**Figure 4:**
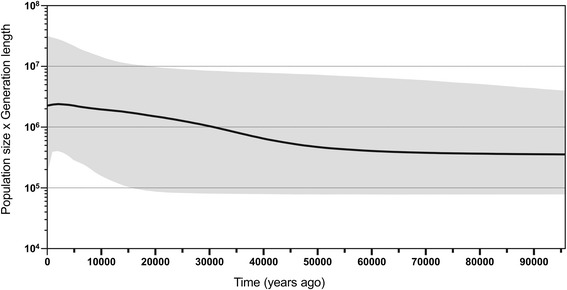
Historical changes in the effective size. Bayesian skyline plot showing the effective population size fluctuation throughout time for *Petunia integrifolia* ssp*. depauperata* (solid line, median estimations; grey area, confidence interval).

The Bayesian Skyline Plot for *P. integrifolia* ssp. *depauperata* may be indicative of smooth yet constant population growth from ~20 000 years ago (Figure 4). This date roughly corresponds to the LGM and is a time when the majority of land was already established in the SACP (the extreme south of this region is considered completely stable only from around 7 000 years ago). This result should be taken with caution given the large credible intervals associated with population size estimates. Interestingly, though, if these estimates are correct, then the signal of population growth exhibited in the neutrality tests may reflect a more recent expansion rather than the original expansion of Southern and Northern groups, which should be older. We also tried to replicate this analysis separately for each haplogroup, but the effective sample sizes were very low, most likely due the lack of enough genetic variation for this estimate.

There are several species endemic to the SACP, but few studies have used a phylogeographic perspective to put in evolutionary context the genetic variation in this region. Mäder et al. [30], studying *C. heterophylla*, found that this species migrated from the west to colonize the SACP, with paleo river channels associated with the main genealogical lineages differentiation at SACP. Lopes et al. [38] studied a subterranean rodent in the same geographic area and identified (modern) rivers acting as effective barriers to gene flow in this species. Strikingly, *P. integrifolia* ssp. *depauperata* seems to be less sensitive to rivers in the SACP, as its genetic groups occurred along a wide area. This may be related to a different seed dispersal efficiency compared with *C. heterophylla*, for which rivers were more efficient barriers [30].

Single-locus studies may be problematic because single markers may tell a different evolutionary history compared to the rest of the genome [39-41]. However, it has also been recently suggested that one or a few small fragments presenting highly informative content could be better in solving the evolutionary relationships among lineages than a large genome survey with incongruent results [42]. In this sense, cpDNA markers applied to *Petunia* and in the closely related genus *Calibrachoa* [33,43] have been able to clarify the evolutionary relationships among species and among lineages within species [24,27,29,44-46].

Considering the results presented here and the absence of many studies in the South Atlantic Coastal Region, especially in regards to plant species and the influence of changes in the sea level on genetic variability distribution, this is an important contribution to the understanding of the processes that drove evolution.

## Conclusion

Climate changes that occurred during the Pleistocene influenced the sea level in the South Atlantic Coastal Plain and promoted genetic differentiation and speciation in the herbaceous annual plant *Petunia integrifolia* ssp. *depauperata*. During this period, plants were restricted to a refuge area, represented by the central part of its current distribution, corresponding to fossil dunes and granitic hills, from which plants colonized the coast as soon as marine regressions exposed suitable lands. This inference is supported by genetic diversity and population expansion statistics. This is the first time that a refuge is proposed on the south edge of the Atlantic Coast.

## Methods

### Plant material, DNA extraction, PCR amplification and sequencing

A total of 291 individuals of *P. integrifolia* ssp. *depauperata* were identified and sampled from 30 locations, with regular distance intervals, covering all of the known distribution of this taxon, including the adjacent northern and southern areas (Figure 1, locations numbered from 1 to 30, hereafter named as populations). In addition, 85 individuals of *P. integrifolia* ssp. *integrifolia* were used in comparative approaches and in dating estimates. We obtained the geographic coordinates by Global Positioning System (GPS), and vouchers were deposited in the BHCB herbarium, Universidade Federal de Minas Gerais, Belo Horizonte, Brazil. Total DNA was extracted from young leaves carefully collected for genetic analysis. Silica gel dried leaves were frozen in liquid nitrogen and ground to a fine powder, and DNA was extracted with cetyltrimethyl ammonium bromide (CTAB) protocol as described by Roy et al. [47]. This work was conducted under permit MP 2.186/16 of the Brazilian Federal Government to access plant genetic information to develop evolutionary or taxonomic studies. No specific collection permits were required because neither taxa are endangered or protected and because no population occurs on protected areas.

Polymerase chain reaction (PCR) was employed to amplify the non-coding plastid *trnH-psbA* and *trnS-trnG* intergenic spacers, using the universal primers previously described ([48,49], respectively), and following amplification conditions described in Lorenz-Lemke et al. [27]. The PCR products were purified according to Dunn and Blatnner [50] and sequenced in a MegaBACE1000 (GE Healthcare Bio Sciences Corp., Piscataway, NY, USA) automatic sequencer according to the manufacturer’s instructions and the DYEnamicET Terminator Sequencing Premix Kit (GE Healthcare). Tables S1 and S2 in Additional information provide voucher information, GenBank accession numbers, and the general geographic information for each sample.

### Sequence analysis and molecular diversity

For each marker, both forward and reverse strands were checked using Chromas (available at website: http://technelysium.com.au/) and aligned manually in GeneDoc [51]. Because the plastid segments are naturally linked and this genome is usually non-recombining, a common situation in plant phylogenies, the two markers were combined in a single concatenated set. All insertion/deletion events (indels) that involved poly A/T were eliminated from the analyses because their homologies cannot be adequately accessed [52]. Contiguous indels of more than one base pair (bp) were treated as one mutational event [53]. Descriptive statistics of genetic variability, such as haplotype (*h*) and nucleotide (*π*) diversities [54], were estimated in Arlequin 3.5 [55].

### Phylogenetic relationships

The evolutionary relationships between haplotypes were estimated using the median-joining network method (e = 0; [56]) as implemented in the Network 4.6 software (available at website: http://www.fluxus-engineering.com/sharenet.htm).

The dated Bayesian phylogenetic tree of haplotypes was inferred using Beast 1.8.0 [57] with a Yule tree prior and the HKY substitution model with four gamma-distributed rate categories based on results from the Akaike Information Criterion in jModelTest 2 [58]. We also used a lognormal relaxed clock and went to the literature for an informed prior for the nucleotide substitution rate. We compiled rates calculated for plastid markers for shrubs or herbaceous plants with generation time of up to three years, features that are similar to *Petunia*. We found eight published rates that fit these criteria, varying between 1 x 10^-9^ and 8.24 x 10^-9^ substitutions per site per year (s/s/y) [59-63]. To take into account this rate heterogeneity, we used a gamma distribution prior with a shape parameter 1.6 and scale parameter 1.6 x 10^-9^ as prior. We assumed an offset value of 1 x 10^-9^ s/s/y, such that the median of the prior was 3.05 x 10^-9^ s/s/y, allowing rate values of 8.24 x 10^-9^ s/s/y to be reached with low probability (distribution graphical result is available in Additional file 3).

### Discrete phylogeographical analysis

For the phylogeographic reconstruction, we modeled geography through the discrete phylogeographic diffusion model of [64] and used the Bayesian stochastic search variable selection (BSSVS) procedure in Beast. We classified sampling locations in four discrete states: southern coastal plain (Southern), northern coastal plain (Northern), Center, and “integrifolia” (corresponding to inland individuals of *P. integrifolia* ssp. *integrifolia*). We then estimated a reconstruction of the phylogeographic history of the clade. We also explored different choices of prior distributions for migration rates in this model, including a uniform prior and a distance-informed prior, and we found that additional information on geographic distances between locations had little impact on the estimated rates.

For each of the above analyses, two independent runs consisting of 1 x 10^8^ Markov chain Monte Carlo (MCMC) iterations were performed, sampling every 1000 generations; 10% of iterations were removed as burn-in. Convergence was checked by visual inspection of the independent runs in Tracer 1.6 [65] so that all parameters had effective sample sizes (ESS) > 200. We obtained the maximum clade credibility (MCC) tree and the posterior probabilities (PP) for each node [66] using the TreeAnnotator from Beast package, and finally, we used Figtree 1.4.0 [67] to draw and edit the resulting phylogenetic trees.

### Geographic structure

We also used Arlequin to perform an analysis of molecular variance (AMOVA; [68]) to quantify the degree of genetic structure among groups of populations, which was performed using Φ-statistics and 1000 permutations to test significance. The Mantel test was used to evaluate the correlation between genetic and geographic distances, using 1000 permutations in the program Alleles in Space 1.0 [69]. We used Samova 1.0 [70] to estimate the number of genetic homogeneous groups (K) for *P. integrifolia* ssp. *depauperata* through a spatial analysis of molecular variance. Following this, we used Arlequin to estimate AMOVA and genetic variability statistics for the groups defined by Samova, as described previously.

### Demographic analysis

We also used Arlequin to perform the neutrality tests Tajima’s D [71] and Fu’s *F*s [72]. Historical changes in the effective size of *P. integrifolia* ssp. *depauperata* were also inferred through the Bayesian Skyline Plot (BSP; [73]) method implemented in Beast, using the same prior and conditions described previously except that all individual sequences were included and the Bayesian Skyline tree prior was used. The BSP reconstruction was done using Tracer 1.6.

### Availability of supporting data

Sequences are deposited in GenBank (accession numbers available in Additional file 1).

## Additional files

Additional file 1: Table S1. Haplotypes information. Additional file 2: Table S2. Sampling information. Additional file 3: Figure S1. Plastid evolutionary rates distribution.
