# Supplementary material for: Were sea level changes during the Pleistocene in the South Atlantic Coastal Plain a driver of speciation in Petunia (Solanaceae)?
Source: BMC Evol Biol. 2015 May 20;15:92. doi: 10.1186/s12862-015-0363-8 (PMC4438590; doi:10.1186/s12862-015-0363-8)
Supplement: Additional file 1: Table S1. — Haplotypes information. [file 12862_2015_363_MOESM1_ESM.doc]

**Additional file 1**

**Table S1.** GenBank numbers

| Haplogroup | Haplotype | Samova group | Population | *trnG-trnS* | *trnH-psbA* |
| --- | --- | --- | --- | --- | --- |
| Center | H1 | SG2 | Pop 1-8 | KP347048 | KP347087 |
| Center | H2 | SG2 | Pop 1 | KP347049 | KP347088 |
| Center | H3 | SG2 | Pop 1 | KP347050 | KP347089 |
| Center | H4 | SG1, SG2 | Pop 1, Pop5-9 | KP347051 | KP347090 |
| Northern | H5 | SG1, SG2, SG3 | Pop7, Pop11, Pop13, Pop 23-30 | KP347052 | KP347091 |
| Northern | H6 | SG3 | Pop29 | KP347053 | KP347092 |
| Northern | H7 | SG3 | Pop23, Pop27-28 | KP347054 | KP347093 |
| Center | H8 | SG2 | Pop2 | KP347055 | KP347094 |
| Center | H9 | SG2 | Pop3 | KP347056 | KP347095 |
| Southern | H10 | SG1 | Pop9-22 | KP347057 | KP347096 |
| Southern | H11 | SG1 | Pop12 | KP347058 | KP347097 |
| Southern | H12 | SG1 | Pop12 | KP347059 | KP347098 |
| Southern | H13 | SG1 | Pop14 | KP347060 | KP347099 |
| Northern | H14 | SG2 | Pop8 | KP347061 | KP347100 |
| Center | H15 | SG2 | Pop4, Pop5 | KP347062 | KP347101 |
| Northern | H16 | SG3 | Pop11 | KP347063 | KP347102 |
| Southern | H17 | SG1 | Pop15, Pop16 | KP347064 | KP347103 |
| Southern | H18 | SG1 | Pop17, Pop21 | KP347065 | KP347104 |
| *** | H19 | - | Pop17 | KP347066 | KP347105 |
| Southern | H20 | SG1 | Pop18 | KP347067 | KP347106 |
| Southern | H21 | SG1 | Pop18 | KP347068 | KP347107 |
| Southern | H22 | SG1 | Pop18 | KP347069 | KP347108 |
| Southern | H23 | SG1 | Pop18 | KP347070 | KP347109 |
| Northern | H24 | SG | Pop26 | KP347071 | KP347110 |
| Northern | H25 | SG | Pop26 | KP347072 | KP347111 |
| Southern | H26 | SG | Pop20 | KP347073 | KP347112 |
| * | H27 | - | - | KP347074 | KP347113 |
| * | H28 | - | - | KP347075 | KP347114 |
| * | H29 | - | - | KP347076 | KP347115 |
| * | H30 | - | - | KP347077 | KP347116 |
| * | H31 | - | - | KP347078 | KP347117 |
| * | H32 | - | - | KP347079 | KP347118 |
| * | H33 | - | - | KP347080 | KP347119 |
| * | H34 | - | - | KP347081 | KP347120 |
| * | H35 | - | - | KP347082 | KP347121 |
| * | H36 | - | - | KP347083 | KP347122 |
| * | H37 | - | - | KP347084 | KP347123 |
| * | H38 | - | - | KP347085 | KP347124 |
| * | H39 | - |  | KP347086 | KP347125 |

* *P.* *integrifolia* ssp. *integrifolia* haplogroup
