# Supplementary material for: Were sea level changes during the Pleistocene in the South Atlantic Coastal Plain a driver of speciation in Petunia (Solanaceae)?
Source: BMC Evol Biol. 2015 May 20;15:92. doi: 10.1186/s12862-015-0363-8 (PMC4438590; doi:10.1186/s12862-015-0363-8)
Supplement: Additional file 2: Table S2. — Sampling information. [file 12862_2015_363_MOESM2_ESM.doc]

**Additional file 2**

**Table S2.** Details of populations (Pop) used in this study; collection site (locality); coordinates; voucher or collector numbers; sample size (n) collected per population; code of cpDNA haplotypes (H) found.

| Taxa | Pop | Locality | Coordinates | Voucher/collector | n | Haplotypes |
| --- | --- | --- | --- | --- | --- | --- |
| *P. integrifolia ssp. depauperata* | 1 | Porto Alegre/RS | 30 04' 28''  51 07' 03'' | BHCB7615 | 07 | H1, H2, H3, H4 |
|  | 2 | Gravataí/RS | 29 56' 34''  50 55' 36'' | BHCB85210 | 04 | H1, H8 |
|  | 3 | S. Antônio da Patrulha/RS | 29 46' 08''  50 34' 23'' | BHCB85214 | 05 | H1, H9 |
|  | 4 | S. Antônio da Patrulha/RS | 29 52'59''  50 32'06'' | BHCB85211 | 09 | H1, H15 |
|  | 5 | S. Antônio da Patrulha/RS | 29 54' 18''  50 25' 34'' | BHCB104837 | 09 | H1, H4, H15 |
|  | 6 | Osório/RS | 29 48' 47''  50 14' 34'' | BHCB104842 | 09 | H1, H4 |
|  | 7 | Capão da Canoa/RS | 29 45' 32''  49 58' 42'' | LEM_pedepa1370 | 14 | H1, H4, H5 |
|  | 8 | Xangrilá/RS | 29 49' 41''  50 03' 59'' | LEM_pedepa1758 | 07 | H1, H4, H14 |
|  | 9 | Tramandaí/RS | 30 00'06''  50 07'50'' | LEM_pedepa131 | 09 | H4, H10 |
|  | 10 | Pinhal/RS | 30 15' 42''  50 14' 09'' | LEM_pedepa73 | 15 | H10 |
|  | 11 | Mostardas/RS | 30 32' 27''  50 25' 10'' | BHCB87278 | 10 | H5, H10, H16 |
|  | 12 | Mostardas/RS | 30 45' 53''  50 37' 35'' | BHCB87281 | 14 | H10, H11, H12 |
|  | 13 | Mostardas/RS | 30 56' 14''  50 44' 21'' | BHCB87283 | 13 | H5, H10 |
|  | 14 | Mostardas/RS | 31 06' 32''  50 54' 04'' | BHCB87285 | 09 | H10, H13 |
|  | 15 | Tavares/RS | 31 14' 15''  51 00' 34'' | BHCB104896 | 11 | H10, H17 |
|  | 16 | S. José do Norte/RS | 31 40' 00''  51 25' 32'' | BHCB104894 | 09 | H10, H17 |
|  | 17 | Rio Grande/RS | 32 07' 31''  52 10' 26'' | BHCB104904 | 09 | H10, H18, H19 |
|  | 18 | Rio Grande/RS | 32 36' 17''  52 29' 30'' | BHCB104843 | 09 | H10, H20, H21, H22, H23 |
|  | 19 | Sta. Vitória do Palmar/RS | 33 38' 13''  53 13' 21'' | BHCB104846 | 07 | H10 |
|  | 20 | La Coronilla/Uruguay | 33 54' 39''  53 30' 44'' | LEM_pedepa1614 | 09 | H10, H26 |
|  | 21 | Pelotas/RS | 31 45'59''  52 15'13'' | BHCB87264 | 08 | H10, H18 |
|  | 22 | S. Lourenço do Sul/RS | 31 22' 36''  51 57' 11'' | BHCB104861 | 09 | H10 |
|  | 23 | Torres/RS | 29 22'53”  49 45'57'' | BHCB79852 | 09 | H5, H7 |
|  | 24 | Gaivota/SC | 29 11' 06''  49 36' 49'' | BHCB79846 | 09 | H5 |
|  | 25 | Arroio do Silva/SC | 28 59' 33''  49 24' 59'' | LEM_pedepa1605 | 08 | H5 |
|  | 26 | Maracajá/SC | 28 50' 31''  49 25' 49'' | LEM_pedepa1591 | 11 | H5, H24, H25 |
|  | 27 | Içara/SC | 28 49' 25''  49 13' 03'' | LEM_pedepa1576 | 15 | H5, H7 |
|  | 28 | Laguna/SC | 28 36' 57''  48 48' 03'' | LEM_pedepa1649 | 11 | H5, H7 |
|  | 29 | Garopaba/SC | 28 01' 17''  48 37' 17'' | BHCB104857 | 12 | H5, H6 |
|  | 30 | Florianópolis/SC | 27 31' 22''  48 25' 01'' | BHCB80104 | 11 | H5 |
| *P. integrifolia spp. integrifolia* | 31 | Viamão/RS | 30 22' 51''  51 00' 17'' | LEM_pedepa1420 | 04 | H27 |
|  | 32 | Barra do Ribeiro/RS | 30 24' 22''  51 12' 09'' | BHCB 85204 | 07 | H27, H30 |
|  | 33 | Guaíba/RS | 30 08' 15''  51 19' 03'' | BHCB104835 | 04 | H27, H28, H29 |
|  | 34 | Cachoeira do Sul/RS | 30 16' 42''  53 07' 04'' | BHCB75139 | 01 | H27 |
|  | 35 | Caçapava do Sul/RS | 30 22' 06''  53 21' 46'' | JRS3158 | 01 | H33 |
|  | 36 | Rio Pardo/RS | 30 04' 21''  52 21' 59'' | BHCB81676 | 01 | H33 |
|  | 37 | Encruzilhada do Sul/RS | 30 22' 08''  52 25' 47'' | JRS3226 | 04 | H27, H38, H39 |
|  | 38 | Cachoeira do Sul/RS | 30 19' 11''  52 55' 34'' | JRS3230 | 01 | H33 |
|  | 39 | Cachoeira do Sul/RS | 30 27' 11''  52 56' 08'' | BHCB75140 | 01 | H37 |
|  | 40 | Cachoeira do Sul/RS | 30 17' 05''  53 08' 00'' | JRS3159 | 02 | H27, H33 |
|  | 41 | Caçapava do Sul/RS | 30 29' 00''  53 22' 04'' | JRS3151 | 02 | H35 |
|  | 42 | Caçapava do Sul/RS | 30 42' 12''  53 33' 24'' | BHCB85209 | 01 | H27 |
|  | 43 | Caçapava do Sul/RS | 30 38' 14''  53 33' 03'' | JRS3174 | 03 | H36 |
|  | 44 | S. Sepé/RS | 30 22' 21''  53 41' 38'' | BHCB79885 | 02 | H27, H33 |
|  | 45 | Caçapava do Sul/RS | 30 22' 19''  53 25' 43'' | BHCB85208 | 02 | H35 |
|  | 46 | Santa Maria/RS | 29 40' 32''  53 57' 55'' | BHCB102085 | 04 | H27 |
|  | 47 | S. Pedro do Sul/RS | 29 38' 05''  54 15' 55'' | BHCB102098 | 03 | H33 |
|  | 48 | S. Pedro do Sul/RS | 29 38' 18''  54 18' 16'' | BHCB102097 | 07 | H33 |
|  | 49 | Alegrete/RS | 29 50' 36''  55 39' 44'' | BHCB79884 | 06 | H33 |
|  | 50 | Alegrete/RS | 29 47' 24''  55 47' 41'' | BHCB85218 | 01 | H33 |
|  | 51 | Quaraí/RS | 30 21' 27''  56 27' 23'' | BHCB79876 | 01 | H27 |
|  | 52 | Quaraí/RS | 30 26' 14''  56 20' 06'' | BHCB102115 | 06 | H27 |
|  | 53 | Quaraí/RS | 30 29' 02''  56 13' 15'' | BHCB102114 | 04 | H27 |
|  | 54 | Santana do Livramento/RS | 30 30' 07''  56 11' 48'' | BHCB102117 | 05 | H27, H34 |
|  | 55 | Santana do Livramento/RS | 30 47' 26''  55 42' 03'' | BHCB79870 | 02 | H32 |
|  | 56 | Santana do Livramento/RS | 30 48' 22''  55 37' 02'' | BHCB79869 | 02 | H32 |
|  | 57 | Santana do Livramento/RS | 30 50' 40''  55 26' 45'' | BHCB79864 | 03 | H31, H32 |
|  | 58 | Santana do Livramento/RS | 30 47' 32''  55 12' 38'' | BHCB79866 | 03 | H27 |
|  | 59 | Santana do Livramento/RS | 30 50' 27''  55 02' 16'' | BHCB79865 | 01 | H31 |
|  | 60 | Dom Pedrito/RS | 30 55' 06''  54 47' 24'' | BHCB79863 | 01 | H31 |

RS (Rio Grande do Sul) SC (Santa Catarina) Brazilian states; BHCB – herbarium of Universidade Federal de Minas Gerais, Belo Horizonte, Brazil; JRS – collector number (João Renato Stehmann); LEM – collector number (Laboratory of Molecular Evolution team)
