# Supplementary figures and images for: Were sea level changes during the Pleistocene in the South Atlantic Coastal Plain a driver of speciation in Petunia (Solanaceae)?
Source: BMC Evol Biol. 2015 May 20;15:92. doi: 10.1186/s12862-015-0363-8 (PMC4438590; doi:10.1186/s12862-015-0363-8)

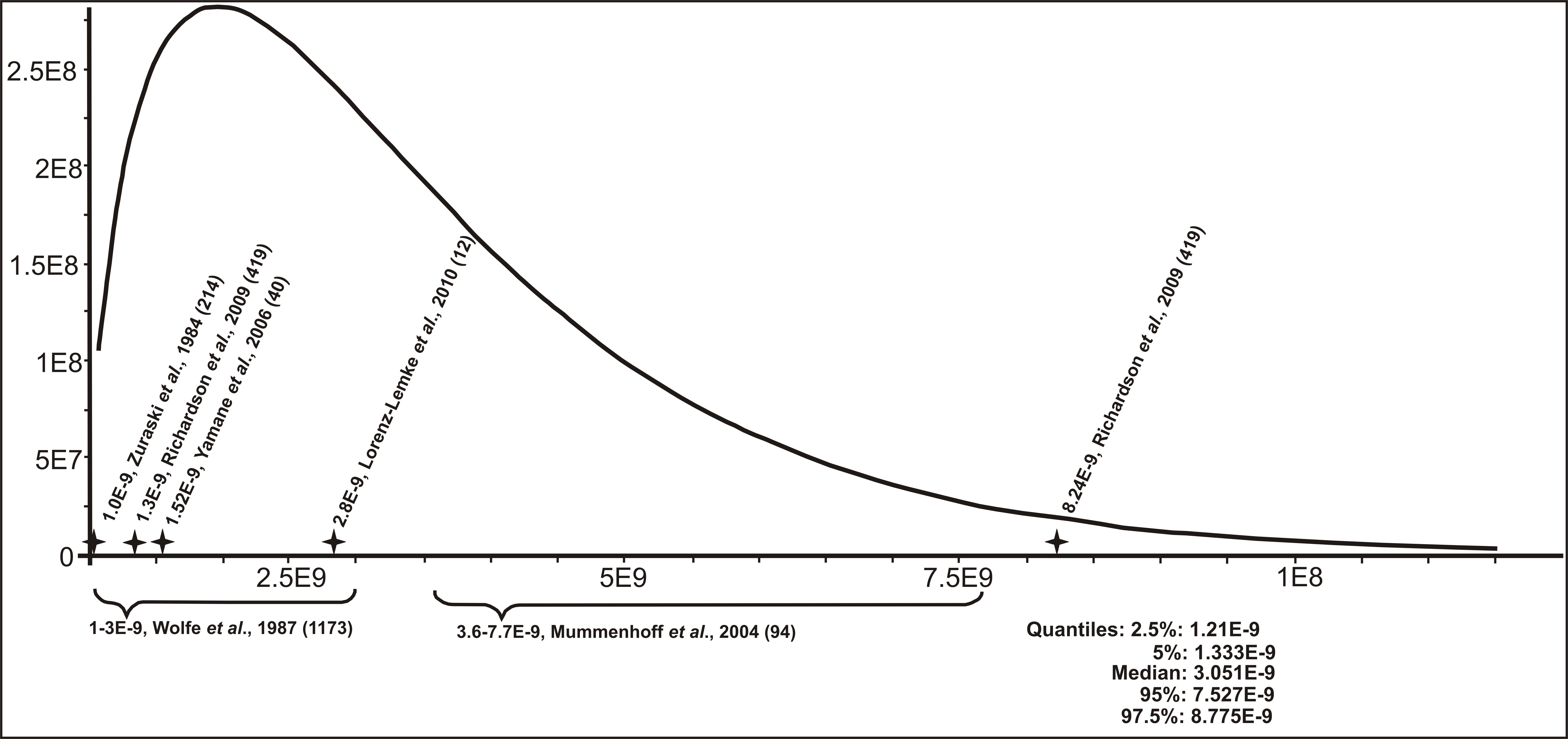

Supplement: Additional file 3: Figure S1. — Plastid evolutionary rates distribution. [file 12862_2015_363_MOESM3_ESM.jpeg]
